# 5-(Adamantan-1-yl)-3-[(4-benzyl­piperazin-1-yl)meth­yl]-1,3,4-oxadiazole-2(3*H*)-thione

**DOI:** 10.1107/S1600536812027249

**Published:** 2012-06-23

**Authors:** Ali A. El-Emam, Nasser R. El-Brollosy, Mohamed I. Attia, Mohammed Said-Abdelbaky, Santiago García-Granda

**Affiliations:** a College of Pharmacy, King Saud University, PO Box 2457, Riyadh 11451, Saudi Arabia; b Departamento de Química Física y Analítica, Facultad de Química, Universidad de Oviedo – CINN, C/ Julián Clavería, 8, 33006 Oviedo, Asturias, Spain

## Abstract

The mol­ecule of the title compound, C_24_H_32_N_4_OS, is a functionalized 1,3,4-oxadiazole-2-thione with substituted piperazine and adamantanyl substituents attached at the 3- and 5-positions, respectively, of the oxadiazole spacer with an approximately C-shaped conformation. In the crystal, mol­ecules form dimers *via* C—H⋯S inter­action. The piperazine ring has a chair conformation; the substituents S, methyl­ene C and adamantane C of the essentially planar oxadiazole ring are approximately in the same plane, with distances of −0.046 (2), −0.085 (5) and 0.003 (4) Å, respectively. The dihedral angle between the planes of the phenyl and oxadiazole rings is 31.3 (3)°.

## Related literature

For the biological activity of adamantyl-1,3,4-oxadiazole derivatives, see: Kadi *et al.* (2007 ▶, 2010 ▶); Al-Deeb *et al.* (2006 ▶), Vernier *et al.* (1969 ▶), El-Emam & Ibrahim (1991 ▶). For the synthesis of the title compound, see: El-Emam *et al.* (2004 ▶). For related adamantane structures, see: Almutairi *et al.* (2012 ▶); Al-Tamimi *et al.* (2010 ▶); Al-Abdullah *et al.* (2012 ▶). For related 1,3,4-oxadiazole structures, see: Fun *et al.* (2011 ▶); El-Emam *et al.* (2012 ▶).
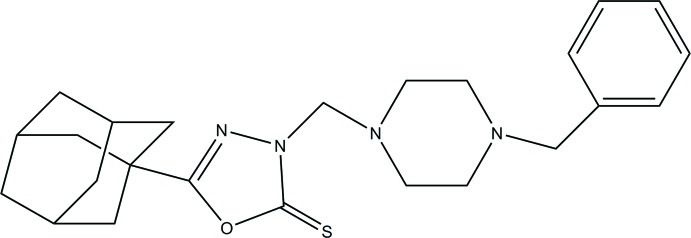


## Experimental

### 

#### Crystal data

C_24_H_32_N_4_OS
*M* *_r_* = 424.61
Monoclinic, 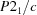

*a* = 11.6417 (9) Å
*b* = 17.198 (2) Å
*c* = 12.774 (1) Å
β = 115.06 (1)°
*V* = 2316.8 (4) Å^3^
*Z* = 4
Cu *K*α radiation
μ = 1.41 mm^−1^
*T* = 293 K
0.11 × 0.09 × 0.02 mm

#### Data collection

Oxford Diffraction Xcalibur Ruby Gemini diffractometer
Absorption correction: multi-scan (*CrysAlis PRO*; Oxford Diffraction, 2010 ▶) *T*_min_ = 0.857, *T*_max_ = 0.975
9969 measured reflections
4368 independent reflections
2049 reflections with *I* > 2σ(*I*)
*R*_int_ = 0.069

#### Refinement

*R*[*F* ^2^ > 2σ(*F* ^2^)] = 0.071
*wR*(*F* ^2^) = 0.219
*S* = 1.01
4368 reflections
271 parameters
H-atom parameters constrained
Δρ_max_ = 0.46 e Å^−3^
Δρ_min_ = −0.22 e Å^−3^

### 

Data collection: *CrysAlis CCD* (Oxford Diffraction, 2010 ▶); cell refinement: *CrysAlis CCD*; data reduction: *CrysAlis RED* (Oxford Diffraction, 2010 ▶); program(s) used to solve structure: *SIR92* (Altomare *et al.*, 1994 ▶); program(s) used to refine structure: *SHELXL97* (Sheldrick, 2008 ▶); molecular graphics: *ORTEP-3 for Windows* (Farrugia, 1997 ▶) and *Mercury* (Macrae *et al.*, 2008 ▶); software used to prepare material for publication: *WinGX* (Farrugia, 1999 ▶), *PLATON* (Spek, 2009 ▶), *PARST95* (Nardelli, 1995 ▶) and *publCIF* (Westrip, 2010 ▶).

## Supplementary Material

Crystal structure: contains datablock(s) global, I. DOI: 10.1107/S1600536812027249/ff2070sup1.cif

Structure factors: contains datablock(s) I. DOI: 10.1107/S1600536812027249/ff2070Isup2.hkl

Supplementary material file. DOI: 10.1107/S1600536812027249/ff2070Isup3.cml

Additional supplementary materials:  crystallographic information; 3D view; checkCIF report

## Figures and Tables

**Table 1:** Hydrogen-bond geometry (Å, °)

*D*—H⋯*A*	*D*—H	H⋯*A*	*D*⋯*A*	*D*—H⋯*A*
C5—H5*B*⋯S1^i^	0.97	2.97	3.652 (5)	128 (1)

Symmetry code: (i) 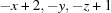.

**Fig. 1.**
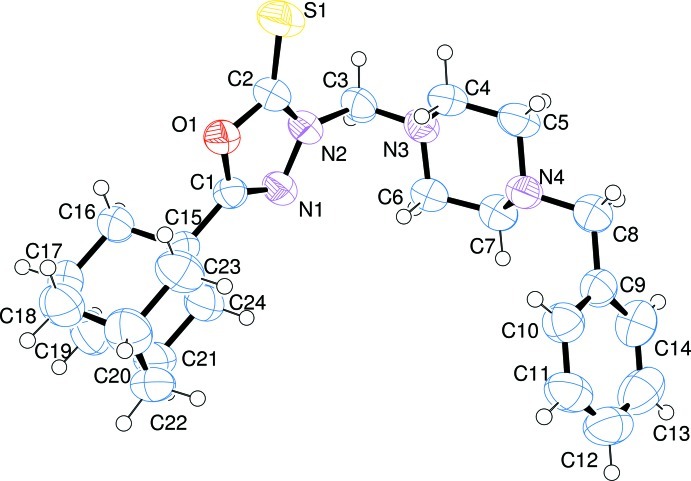
An ORTEP-style plot of title compound with labeling. Ellipsoids are given at the 50% probability level.

## supplementary crystallographic information

### Comment

Considerable attention has been devoted to adamantane derivatives, which have been known for their diverse biological properties as antiviral against the influenza (Vernier *et al.*, 1969) and HIV viruses (El-Emam *et al.*, 2004). Moreover, adamantane derivatives were recently reported to exhibit remarkable antibacterial (Kadi *et al.*, 2007, 2010) and anti-inflammatory (El-Emam & Ibrahim, 1991) activities. In an earlier publication (El-Emam *et al.*, 2004), we reported the synthesis and potent antimicrobial and antiviral activities of a series of 1-adamantyl-1,3,4-oxadiazoles and related derivatives including the title compound (I).

Molecules of the title compound form dimers connected to each other through C5—H5B···S1 with distance 3.652 (4) Å and bond angle 128.2 (3)°. The planar oxadiazole ring includes S1, C3 and C15 approximately in the same plane with distances -0.046 (2) Å, -0.085 (5) Å and 0.003 (4) Å respectively. The plane defined by the phenyl ring contains C8 with distance 0.021 (5) A°. These planar structures do not deviate significantly from planarity and the dihedral angle between the two planes is 31.3 (3)°.

### Experimental

A mixture of 5-(Adamantan-1-yl)-1,3,4-oxadiazole-2-thiol (2.36 g, 0.01 mol), *N*-benzylpiperazine (1.76 g, 0.01 mol) and 37% formaldehyde solution (1.5 ml), in ethanol (15 ml), was stirred at room temperature for 2 h. and allowed to stand overnight. The precipitated crude product was filtered, washed with water, dried, and crystallized from ethanol to yield 3.18 g (75%) of the title compound I (C_24_H_32_N_4_OS) as fine colorless needles crystals. *M*.p. 127–129 °C. Single crystals suitable for X-ray analysis were obtained by slow evaporation of the compound solution in chloroform-ethanol (1:1; 10 ml) at room temperature. ^1^H NMR (CDCl_3_, 500.13 MHz): δ 1.72–1.75 (m, 6H, Adamantane-H), 1.99 (s, 6H, Adamantane-H), 2.11 (s, 3H, Adamantane-H), 2.49 (t, 4H, Piperazine-CH_2_), 2.85 (t, 4H, Piperazine-CH_2_), 3.52 (s, 2H, CH_2_Ph), 4.98 (s, 2H, CH_2_), 7.25–7.34 (m, 5H, Ar—H). ^13^C NMR (CDCl_3_, 125.76 MHz): δ 27.48, 34.36, 36.11, 39.11 (Adamantane-C), 50.20, 52.94 (Piperazine-C), 63.14 (CH_2_Ph), 69.99 (CH_2_), 127.16, 128.16, 129.29, 137.74 (Ar—C), 167.76 (Oxadiazole C=N), 178.62 (C=S).

### Refinement

Carbon-bound H-atoms were placed in calculated positions (C—H 0.95 to 0.98 Å) and were included in the refinement in the riding model approximation, with U(H) set to 1.2 to 1.5U(C). At the end of the refinement the highest peak in the electron density was 0.4600 e Å ^-3^, while the deepest hole was -0.2200 e Å ^-3^.

### Crystal data

**Table:**

C_24_H_32_N_4_OS	*F*(000) = 912
*M**_r_* = 424.61	*D*_x_ = 1.217 Mg m^−^^3^
Monoclinic, *P*2_1_/*c*	Cu *K*α radiation, λ = 1.54180 Å
Hall symbol: -P 2ybc	Cell parameters from 1030 reflections
*a* = 11.6417 (9) Å	θ = 3.8–70.6°
*b* = 17.198 (2) Å	µ = 1.41 mm^−^^1^
*c* = 12.774 (1) Å	*T* = 293 K
β = 115.06 (1)°	Prismatic, colourless
*V* = 2316.8 (4) Å^3^	0.11 × 0.09 × 0.02 mm
*Z* = 4	

### Data collection

**Table:**

Oxford Diffraction Xcalibur Ruby Gemini diffractometer	4368 independent reflections
Radiation source: fine-focus sealed tube	2049 reflections with *I* > 2σ(*I*)
Graphite monochromator	*R*_int_ = 0.069
Detector resolution: 10.2673 pixels mm^-1^	θ_max_ = 70.7°, θ_min_ = 4.2°
ω scans	*h* = −14→13
Absorption correction: multi-scan (*CrysAlis PRO*; Oxford Diffraction, 2010)	*k* = −17→21
*T*_min_ = 0.857, *T*_max_ = 0.975	*l* = −15→15
9969 measured reflections	

### Refinement

**Table:**

Refinement on *F*^2^	Primary atom site location: structure-invariant direct methods
Least-squares matrix: full	Secondary atom site location: difference Fourier map
*R*[*F*^2^ > 2σ(*F*^2^)] = 0.071	Hydrogen site location: inferred from neighbouring sites
*wR*(*F*^2^) = 0.219	H-atom parameters constrained
*S* = 1.01	*w* = 1/[σ^2^(*F*_o_^2^) + (0.0727*P*)^2^] where *P* = (*F*_o_^2^ + 2*F*_c_^2^)/3
4368 reflections	(Δ/σ)_max_ < 0.001
271 parameters	Δρ_max_ = 0.46 e Å^−^^3^
0 restraints	Δρ_min_ = −0.22 e Å^−^^3^

### Special details

**Table:**

Experimental. Absorption correction: CrysAlis PRO (Oxford Diffraction, 2010) Empirical absorption correction using spherical harmonics, implemented in SCALE3 ABSPACK scaling algorithm.
Geometry. All e.s.d.'s (except the e.s.d. in the dihedral angle between two l.s. planes) are estimated using the full covariance matrix. The cell e.s.d.'s are taken into account individually in the estimation of e.s.d.'s in distances, angles and torsion angles; correlations between e.s.d.'s in cell parameters are only used when they are defined by crystal symmetry. An approximate (isotropic) treatment of cell e.s.d.'s is used for estimating e.s.d.'s involving l.s. planes.
Refinement. Refinement of *F*^2^ against ALL reflections. The weighted *R*-factor *wR* and goodness of fit *S* are based on *F*^2^, conventional *R*-factors *R* are based on *F*, with *F* set to zero for negative *F*^2^. The threshold expression of *F*^2^ > σ(*F*^2^) is used only for calculating *R*-factors(gt) *etc*. and is not relevant to the choice of reflections for refinement. *R*-factors based on *F*^2^ are statistically about twice as large as those based on *F*, and *R*- factors based on ALL data will be even larger.

### Fractional atomic coordinates and isotropic or equivalent isotropic displacement parameters (Å^2^)

**Table:**

	*x*	*y*	*z*	*U*_iso_*/*U*_eq_	
S1	0.87513 (13)	0.09736 (11)	0.58610 (13)	0.1011 (6)	
O1	0.6438 (3)	0.15777 (18)	0.4778 (2)	0.0621 (8)	
N3	0.8803 (3)	0.1628 (2)	0.2778 (3)	0.0581 (9)	
N4	0.8057 (3)	0.0547 (2)	0.0914 (3)	0.0577 (9)	
N2	0.7676 (3)	0.1910 (2)	0.3997 (3)	0.0588 (9)	
N1	0.6514 (3)	0.2280 (2)	0.3359 (3)	0.0579 (9)	
C1	0.5809 (4)	0.2066 (2)	0.3858 (3)	0.0502 (9)	
C15	0.4469 (3)	0.2263 (2)	0.3571 (3)	0.0489 (9)	
C6	0.7735 (4)	0.1783 (3)	0.1668 (4)	0.0606 (11)	
H6A	0.7655	0.2339	0.1527	0.073*	
H6B	0.6957	0.1599	0.1688	0.073*	
C9	0.6932 (4)	0.0165 (3)	−0.1133 (4)	0.0623 (11)	
C7	0.7926 (4)	0.1384 (3)	0.0711 (4)	0.0639 (11)	
H7A	0.7208	0.1488	−0.0020	0.077*	
H7B	0.8682	0.1586	0.0667	0.077*	
C5	0.9163 (4)	0.0404 (3)	0.2003 (4)	0.0670 (12)	
H5A	0.9917	0.0603	0.1952	0.080*	
H5B	0.9271	−0.0152	0.2143	0.080*	
C4	0.9009 (4)	0.0790 (3)	0.2991 (4)	0.0612 (11)	
H4A	0.8293	0.0564	0.3080	0.073*	
H4B	0.9763	0.0704	0.3701	0.073*	
C16	0.4394 (4)	0.2710 (3)	0.4570 (4)	0.0722 (13)	
H16A	0.4883	0.3186	0.4707	0.087*	
H16B	0.4749	0.2398	0.5268	0.087*	
C2	0.7658 (4)	0.1489 (3)	0.4861 (4)	0.0637 (11)	
C8	0.8150 (4)	0.0136 (3)	−0.0047 (4)	0.0712 (13)	
H8A	0.8372	−0.0403	0.0170	0.085*	
H8B	0.8825	0.0366	−0.0200	0.085*	
C3	0.8766 (4)	0.2065 (3)	0.3718 (4)	0.0651 (12)	
H3A	0.8764	0.2613	0.3541	0.078*	
H3B	0.9540	0.1961	0.4402	0.078*	
C21	0.2521 (4)	0.2984 (4)	0.2190 (4)	0.0848 (16)	
H21	0.2164	0.3313	0.1499	0.102*	
C17	0.2980 (5)	0.2908 (3)	0.4275 (4)	0.0810 (15)	
H17	0.2919	0.3189	0.4917	0.097*	
C14	0.6870 (5)	0.0445 (3)	−0.2163 (4)	0.0781 (14)	
H14	0.7604	0.0629	−0.2196	0.094*	
C24	0.3926 (4)	0.2767 (4)	0.2486 (4)	0.0810 (15)	
H24A	0.3967	0.2486	0.1844	0.097*	
H24B	0.4427	0.3237	0.2610	0.097*	
C10	0.5819 (4)	−0.0092 (3)	−0.1108 (4)	0.0698 (12)	
H10	0.5831	−0.0273	−0.0417	0.084*	
C19	0.2511 (5)	0.3400 (3)	0.3222 (5)	0.0881 (16)	
H19A	0.3037	0.3861	0.3374	0.106*	
H19B	0.1653	0.3566	0.3046	0.106*	
C22	0.1798 (5)	0.2217 (4)	0.1996 (5)	0.098 (2)	
H22A	0.0905	0.2324	0.1760	0.118*	
H22B	0.1883	0.1931	0.1377	0.118*	
C11	0.4697 (5)	−0.0085 (3)	−0.2086 (5)	0.0816 (15)	
H11	0.3960	−0.0268	−0.2055	0.098*	
C12	0.4655 (6)	0.0190 (3)	−0.3111 (5)	0.0928 (18)	
H12	0.3895	0.0193	−0.3773	0.111*	
C23	0.3697 (4)	0.1522 (3)	0.3371 (5)	0.0806 (15)	
H23A	0.4032	0.1204	0.4064	0.097*	
H23B	0.3751	0.1226	0.2747	0.097*	
C20	0.2280 (5)	0.1735 (4)	0.3056 (5)	0.0895 (17)	
H20	0.1774	0.1259	0.2921	0.107*	
C18	0.2269 (5)	0.2162 (4)	0.4060 (5)	0.0908 (17)	
H18A	0.2641	0.1837	0.4743	0.109*	
H18B	0.1398	0.2269	0.3922	0.109*	
C13	0.5746 (7)	0.0460 (3)	−0.3148 (5)	0.0962 (19)	
H13	0.5727	0.0652	−0.3835	0.115*	

### Atomic displacement parameters (Å^2^)

**Table:**

	*U*^11^	*U*^22^	*U*^33^	*U*^12^	*U*^13^	*U*^23^
S1	0.0724 (8)	0.1413 (14)	0.0814 (9)	0.0392 (9)	0.0245 (7)	0.0333 (9)
O1	0.0553 (16)	0.078 (2)	0.0531 (16)	0.0100 (15)	0.0228 (13)	0.0091 (15)
N3	0.0456 (17)	0.062 (2)	0.065 (2)	0.0015 (16)	0.0219 (16)	−0.0016 (18)
N4	0.0491 (17)	0.060 (2)	0.058 (2)	0.0056 (16)	0.0174 (16)	0.0011 (17)
N2	0.0415 (17)	0.071 (2)	0.063 (2)	0.0038 (16)	0.0215 (15)	0.0008 (18)
N1	0.0446 (17)	0.066 (2)	0.063 (2)	0.0033 (16)	0.0225 (16)	0.0017 (18)
C1	0.046 (2)	0.053 (2)	0.047 (2)	−0.0005 (18)	0.0147 (17)	−0.0036 (18)
C15	0.0444 (19)	0.056 (2)	0.044 (2)	−0.0053 (17)	0.0167 (16)	−0.0058 (18)
C6	0.052 (2)	0.062 (3)	0.063 (3)	0.005 (2)	0.020 (2)	0.005 (2)
C9	0.061 (3)	0.063 (3)	0.063 (3)	−0.001 (2)	0.026 (2)	−0.006 (2)
C7	0.058 (2)	0.064 (3)	0.069 (3)	0.002 (2)	0.027 (2)	0.009 (2)
C5	0.048 (2)	0.061 (3)	0.077 (3)	0.003 (2)	0.012 (2)	−0.003 (2)
C4	0.045 (2)	0.067 (3)	0.060 (3)	0.0044 (19)	0.0115 (18)	0.004 (2)
C16	0.050 (2)	0.096 (4)	0.066 (3)	0.001 (2)	0.021 (2)	−0.016 (3)
C2	0.047 (2)	0.076 (3)	0.062 (3)	0.009 (2)	0.018 (2)	0.001 (2)
C8	0.055 (2)	0.084 (3)	0.072 (3)	0.001 (2)	0.025 (2)	−0.006 (3)
C3	0.041 (2)	0.081 (3)	0.072 (3)	−0.005 (2)	0.023 (2)	−0.009 (2)
C21	0.058 (3)	0.129 (5)	0.061 (3)	0.007 (3)	0.020 (2)	0.020 (3)
C17	0.076 (3)	0.105 (4)	0.070 (3)	0.003 (3)	0.037 (3)	−0.015 (3)
C14	0.099 (4)	0.068 (3)	0.078 (3)	−0.008 (3)	0.047 (3)	0.001 (3)
C24	0.064 (3)	0.110 (4)	0.070 (3)	0.018 (3)	0.029 (2)	0.020 (3)
C10	0.064 (3)	0.074 (3)	0.070 (3)	−0.004 (2)	0.028 (2)	0.001 (2)
C19	0.083 (3)	0.085 (4)	0.103 (4)	0.019 (3)	0.046 (3)	0.002 (3)
C22	0.062 (3)	0.140 (5)	0.070 (3)	0.017 (3)	0.005 (3)	−0.025 (4)
C11	0.059 (3)	0.074 (3)	0.096 (4)	−0.003 (2)	0.018 (3)	−0.008 (3)
C12	0.088 (4)	0.073 (3)	0.083 (4)	0.010 (3)	0.002 (3)	−0.015 (3)
C23	0.061 (3)	0.071 (3)	0.101 (4)	−0.010 (2)	0.025 (3)	−0.005 (3)
C20	0.072 (3)	0.091 (4)	0.107 (5)	−0.023 (3)	0.038 (3)	−0.016 (4)
C18	0.064 (3)	0.123 (5)	0.089 (4)	−0.004 (3)	0.035 (3)	0.012 (4)
C13	0.134 (5)	0.077 (4)	0.069 (4)	0.009 (4)	0.034 (4)	0.004 (3)

### Geometric parameters (Å, º)

**Table:**

S1—C2	1.631 (4)	C8—H8B	0.9700
O1—C1	1.376 (5)	C3—H3A	0.9700
O1—C2	1.387 (5)	C3—H3B	0.9700
N3—C3	1.432 (6)	C21—C19	1.504 (8)
N3—C6	1.461 (5)	C21—C22	1.528 (9)
N3—C4	1.468 (6)	C21—C24	1.560 (7)
N4—C7	1.459 (6)	C21—H21	0.9800
N4—C8	1.460 (6)	C17—C19	1.484 (8)
N4—C5	1.462 (5)	C17—C18	1.487 (8)
N2—C2	1.327 (6)	C17—H17	0.9800
N2—N1	1.403 (5)	C14—C13	1.379 (8)
N2—C3	1.480 (5)	C14—H14	0.9300
N1—C1	1.287 (5)	C24—H24A	0.9700
C1—C15	1.483 (5)	C24—H24B	0.9700
C15—C23	1.518 (6)	C10—C11	1.374 (6)
C15—C16	1.524 (6)	C10—H10	0.9300
C15—C24	1.526 (6)	C19—H19A	0.9700
C6—C7	1.498 (6)	C19—H19B	0.9700
C6—H6A	0.9700	C22—C20	1.481 (8)
C6—H6B	0.9700	C22—H22A	0.9700
C9—C14	1.372 (7)	C22—H22B	0.9700
C9—C10	1.382 (6)	C11—C12	1.373 (8)
C9—C8	1.508 (6)	C11—H11	0.9300
C7—H7A	0.9700	C12—C13	1.371 (8)
C7—H7B	0.9700	C12—H12	0.9300
C5—C4	1.503 (6)	C23—C20	1.566 (7)
C5—H5A	0.9700	C23—H23A	0.9700
C5—H5B	0.9700	C23—H23B	0.9700
C4—H4A	0.9700	C20—C18	1.483 (8)
C4—H4B	0.9700	C20—H20	0.9800
C16—C17	1.562 (7)	C18—H18A	0.9700
C16—H16A	0.9700	C18—H18B	0.9700
C16—H16B	0.9700	C13—H13	0.9300
C8—H8A	0.9700		
			
C1—O1—C2	106.8 (3)	N2—C3—H3B	108.2
C3—N3—C6	113.7 (3)	H3A—C3—H3B	107.4
C3—N3—C4	114.8 (4)	C19—C21—C22	110.2 (5)
C6—N3—C4	111.3 (3)	C19—C21—C24	107.3 (4)
C7—N4—C8	111.5 (4)	C22—C21—C24	106.3 (5)
C7—N4—C5	108.6 (3)	C19—C21—H21	111.0
C8—N4—C5	111.7 (3)	C22—C21—H21	111.0
C2—N2—N1	112.3 (3)	C24—C21—H21	111.0
C2—N2—C3	128.0 (4)	C19—C17—C18	111.2 (4)
N1—N2—C3	119.5 (4)	C19—C17—C16	106.8 (4)
C1—N1—N2	104.0 (3)	C18—C17—C16	107.7 (5)
N1—C1—O1	112.2 (3)	C19—C17—H17	110.4
N1—C1—C15	129.5 (4)	C18—C17—H17	110.4
O1—C1—C15	118.3 (3)	C16—C17—H17	110.4
C1—C15—C23	109.6 (4)	C9—C14—C13	121.7 (5)
C1—C15—C16	109.7 (3)	C9—C14—H14	119.1
C23—C15—C16	108.9 (4)	C13—C14—H14	119.1
C1—C15—C24	110.0 (3)	C15—C24—C21	110.2 (4)
C23—C15—C24	109.6 (4)	C15—C24—H24A	109.6
C16—C15—C24	109.0 (4)	C21—C24—H24A	109.6
N3—C6—C7	110.4 (4)	C15—C24—H24B	109.6
N3—C6—H6A	109.6	C21—C24—H24B	109.6
C7—C6—H6A	109.6	H24A—C24—H24B	108.1
N3—C6—H6B	109.6	C11—C10—C9	121.0 (5)
C7—C6—H6B	109.6	C11—C10—H10	119.5
H6A—C6—H6B	108.1	C9—C10—H10	119.5
C14—C9—C10	117.7 (4)	C17—C19—C21	112.8 (5)
C14—C9—C8	122.7 (5)	C17—C19—H19A	109.0
C10—C9—C8	119.5 (4)	C21—C19—H19A	109.0
N4—C7—C6	110.3 (4)	C17—C19—H19B	109.0
N4—C7—H7A	109.6	C21—C19—H19B	109.0
C6—C7—H7A	109.6	H19A—C19—H19B	107.8
N4—C7—H7B	109.6	C20—C22—C21	111.5 (4)
C6—C7—H7B	109.6	C20—C22—H22A	109.3
H7A—C7—H7B	108.1	C21—C22—H22A	109.3
N4—C5—C4	110.7 (4)	C20—C22—H22B	109.3
N4—C5—H5A	109.5	C21—C22—H22B	109.3
C4—C5—H5A	109.5	H22A—C22—H22B	108.0
N4—C5—H5B	109.5	C12—C11—C10	120.4 (5)
C4—C5—H5B	109.5	C12—C11—H11	119.8
H5A—C5—H5B	108.1	C10—C11—H11	119.8
N3—C4—C5	110.3 (4)	C13—C12—C11	119.3 (5)
N3—C4—H4A	109.6	C13—C12—H12	120.3
C5—C4—H4A	109.6	C11—C12—H12	120.3
N3—C4—H4B	109.6	C15—C23—C20	109.3 (4)
C5—C4—H4B	109.6	C15—C23—H23A	109.8
H4A—C4—H4B	108.1	C20—C23—H23A	109.8
C15—C16—C17	109.7 (3)	C15—C23—H23B	109.8
C15—C16—H16A	109.7	C20—C23—H23B	109.8
C17—C16—H16A	109.7	H23A—C23—H23B	108.3
C15—C16—H16B	109.7	C22—C20—C18	112.0 (5)
C17—C16—H16B	109.7	C22—C20—C23	108.4 (5)
H16A—C16—H16B	108.2	C18—C20—C23	106.7 (5)
N2—C2—O1	104.7 (3)	C22—C20—H20	109.9
N2—C2—S1	131.7 (3)	C18—C20—H20	109.9
O1—C2—S1	123.6 (4)	C23—C20—H20	109.9
N4—C8—C9	112.4 (4)	C20—C18—C17	112.2 (5)
N4—C8—H8A	109.1	C20—C18—H18A	109.2
C9—C8—H8A	109.1	C17—C18—H18A	109.2
N4—C8—H8B	109.1	C20—C18—H18B	109.2
C9—C8—H8B	109.1	C17—C18—H18B	109.2
H8A—C8—H8B	107.9	H18A—C18—H18B	107.9
N3—C3—N2	116.2 (3)	C12—C13—C14	119.8 (6)
N3—C3—H3A	108.2	C12—C13—H13	120.1
N2—C3—H3A	108.2	C14—C13—H13	120.1
N3—C3—H3B	108.2		
			
C2—N2—N1—C1	−0.5 (5)	C4—N3—C3—N2	−67.1 (5)
C3—N2—N1—C1	−175.9 (4)	C2—N2—C3—N3	102.8 (5)
N2—N1—C1—O1	−0.1 (4)	N1—N2—C3—N3	−82.5 (5)
N2—N1—C1—C15	−179.5 (4)	C15—C16—C17—C19	−60.8 (6)
C2—O1—C1—N1	0.7 (4)	C15—C16—C17—C18	58.7 (6)
C2—O1—C1—C15	−179.9 (3)	C10—C9—C14—C13	−1.0 (8)
N1—C1—C15—C23	123.4 (5)	C8—C9—C14—C13	179.4 (5)
O1—C1—C15—C23	−55.9 (5)	C1—C15—C24—C21	−179.3 (4)
N1—C1—C15—C16	−117.1 (5)	C23—C15—C24—C21	60.1 (6)
O1—C1—C15—C16	63.5 (5)	C16—C15—C24—C21	−59.0 (5)
N1—C1—C15—C24	2.8 (6)	C19—C21—C24—C15	58.0 (6)
O1—C1—C15—C24	−176.5 (4)	C22—C21—C24—C15	−59.9 (6)
C3—N3—C6—C7	172.9 (4)	C14—C9—C10—C11	1.5 (7)
C4—N3—C6—C7	−55.5 (5)	C8—C9—C10—C11	−178.9 (5)
C8—N4—C7—C6	175.8 (3)	C18—C17—C19—C21	−54.3 (6)
C5—N4—C7—C6	−60.7 (5)	C16—C17—C19—C21	62.9 (6)
N3—C6—C7—N4	58.8 (5)	C22—C21—C19—C17	53.6 (6)
C7—N4—C5—C4	60.2 (5)	C24—C21—C19—C17	−61.7 (6)
C8—N4—C5—C4	−176.4 (4)	C19—C21—C22—C20	−53.4 (6)
C3—N3—C4—C5	−174.3 (3)	C24—C21—C22—C20	62.6 (6)
C6—N3—C4—C5	54.7 (5)	C9—C10—C11—C12	−1.0 (8)
N4—C5—C4—N3	−57.3 (5)	C10—C11—C12—C13	−0.2 (9)
C1—C15—C16—C17	−179.2 (4)	C1—C15—C23—C20	−179.4 (4)
C23—C15—C16—C17	−59.3 (5)	C16—C15—C23—C20	60.7 (5)
C24—C15—C16—C17	60.3 (5)	C24—C15—C23—C20	−58.5 (6)
N1—N2—C2—O1	0.9 (5)	C21—C22—C20—C18	54.6 (6)
C3—N2—C2—O1	175.8 (4)	C21—C22—C20—C23	−62.8 (6)
N1—N2—C2—S1	−178.2 (4)	C15—C23—C20—C22	59.6 (6)
C3—N2—C2—S1	−3.3 (8)	C15—C23—C20—C18	−61.2 (6)
C1—O1—C2—N2	−0.9 (4)	C22—C20—C18—C17	−55.2 (6)
C1—O1—C2—S1	178.3 (3)	C23—C20—C18—C17	63.3 (6)
C7—N4—C8—C9	−67.9 (5)	C19—C17—C18—C20	54.3 (6)
C5—N4—C8—C9	170.4 (4)	C16—C17—C18—C20	−62.4 (6)
C14—C9—C8—N4	124.2 (5)	C11—C12—C13—C14	0.7 (9)
C10—C9—C8—N4	−55.3 (6)	C9—C14—C13—C12	−0.1 (9)
C6—N3—C3—N2	62.8 (5)		

### Hydrogen-bond geometry (Å, º)

**Table:**

*D*—H···*A*	*D*—H	H···*A*	*D*···*A*	*D*—H···*A*
C5—H5*B*···S1^i^	0.97	2.97	3.652 (5)	128 (1)

Symmetry code: (i) −*x*+2, −*y*, −*z*+1.
